# Artificial Intelligence and Machine Learning in Diagnostic Pathology: A Systematic Review of Applications, Challenges, and Clinical Implications

**DOI:** 10.7759/cureus.105167

**Published:** 2026-03-13

**Authors:** Vartika Mishra, Santosh Jayant, Ankita Dash, Chintan Chaudhary, Sneha Samir Babaria

**Affiliations:** 1 Department of Pathology, Ruxmaniben Deepchand Gardi Medical College, Madhya Pradesh Medical Science University, Ujjain, IND; 2 Department of Pathology, Amaltas University, Bangar, IND; 3 Department of Pathology, Government Medical College and Hospital, Datia, Madhya Pradesh Medical Science University, Datia, IND; 4 Department of Orthopaedics, GMERS (Gujarat Medical Education and Research Society) Medical College and Hospital, Sola, IND; 5 Department of Pathology, Dr. ND Desai Faculty of Medical Science and Research, Dharmsinh Desai University, Nadiad, IND

**Keywords:** artificial intelligence, cytopathology, deep learning, diagnostic pathology, digital pathology, machine learning, neurodegenerative diseases, tumor classification, tumor staging, tumour detection

## Abstract

Artificial intelligence (AI) and machine learning (ML) are transforming diagnostic medicine, particularly in pathology, where image-based interpretation is central to clinical decision-making. This systematic review aimed to examine recent advances, performance outcomes, and practical challenges associated with incorporating AI and ML into diagnostic pathology. A comprehensive literature search was conducted across major scientific databases for studies published between 2010 and 2025. Titles and abstracts were screened independently, full texts were assessed against predefined eligibility criteria, and data were extracted using standardised procedures. Methodological quality was evaluated using established critical appraisal tools appropriate to study design, with structured risk-of-bias assessment reported for diagnostic accuracy studies. A total of 13 studies fulfilled the inclusion criteria, covering multiple pathological domains including breast pathology, cytopathology, neuropathology, head and neck oncology, and multi-organ computational pathology. Across the included studies, deep learning approaches demonstrated high diagnostic performance for tumour detection, classification, and staging tasks. While several investigations incorporated external validation, most were retrospective in design and relied on secondary datasets. Risk-of-bias assessment indicated predominantly moderate overall risk, primarily related to study design and applicability concerns. The evidence suggests that AI and ML systems demonstrate strong technical performance in controlled validation settings and may function as assistive tools within digital pathology workflows.

## Introduction and background

Machine learning (ML) and artificial intelligence (AI) are emerging disruptive technologies in the healthcare sector, which can transform the diagnostic procedure, treatment strategy, and prognostics [1]. These forms of computation are particularly useful in cases where imaging and pattern recognition in significant quantities are at stake, such as in diagnostic radiology, oncology, and pathology [2]. The AI systems can assist clinicians with their working process, simulating human cognition and learning on the basis of complicated data and aiding in different procedures, such as the diagnosis of diseases and their treatment choice, thereby making the work more accurate and efficient [3]. Over the past 10 years, medical imaging and oncology have been the areas of AI and ML that have enjoyed the most publicity, with the deep learning algorithms being the most effective in cancer imaging and staging [4]. As an example, convolutional neural networks (CNNs) and radiomics are effective in the detection and treatment planning of lung cancer [5]. On the same note, AI-based frameworks have also shown promise in biomarker discovery and in making therapeutic decisions in cancer research [6]. These are remarkable advances, but they indicate the possibility of AI to increase diagnostic skills in numerous fields of medicine [7].

Beyond oncology, AI has also been applied in areas such as neurodegenerative diseases and biomarker discovery. In neurological diagnostics, some of the most difficult tasks have been solved with the help of algorithms: the use of ML methods to integrate imaging biomarkers in early Alzheimer diagnosis [8]. Similarly, deep learning frameworks are increasingly used in medical image analysis in a wide variety of fields, heralding a paradigm shift in the way clinicians interact with imaging data [9]. Predictive algorithms are also being developed in laboratory medicine and are already being implemented into routine laboratory workflows to increase diagnostic efficiency [10]. Radiomics in breast cancer also shows the translational value of AI and where imaging-based biomarkers are gaining popularity to help with personalised treatment planning [11].

In cancer, AI/ML has been helpful in pre-operative staging and prognostication. As an example, AI models were used in colorectal cancer to preoperatively stage lymph nodes with clinically relevant accuracy [12]. Moreover, the broader scope of AI in medicine and pathology underscores the importance of AI in shaping future diagnostic processes, with ML being described as an indispensable aspect of enhancing the accuracy and efficiency of diagnosis [13]. The creation of pathology foundation models that combine multimodal data to predict not only the diagnosis of cancer, but also its prognosis, is a step in this transformation [14].

Adequacy of AI systems has also been proven by meta-analysis and systematic reviews. Deep learning and radiomics have been applied to lung cancer staging, which has been shown to perform as well as human experts [15]. Much the same, ML-based methods have also been used to discover new diagnostic markers, such as in colorectal cancer by using exosomal proteomic signatures [16]. In breast cancer pathology, AI has proven to be very useful in increasing the sensitivity of the detection and reproducibility of the workflow [14]. This is also supported by the research on deep learning in breast cancer imaging that reports not only the accuracy of diagnosis but also efficiency improvements [17]. AI-based models of prognostication have also grown to include survival prediction, including lung malignancy on the basis of PET/CT imaging [18]. In prostate cancer, MRI is also seeing deep learning-based applications that allow a more precise diagnosis [19]. This is not limited to oncology, and ML is also applied to other clinical fields, including orthodontics, where it has been applied to segment cone-beam CTs [20]. Comparably, bioinformatics-based ML pipelines have been used to find biomarkers in hepatocellular carcinoma diagnostics [21]. Although these examples demonstrate the broad applicability of AI in medicine, they primarily illustrate the technological foundations that have facilitated the development of AI applications in digital pathology and histopathological image analysis.

AI is not restricted to oncology and neurology only, but also to infectious diseases and cardiovascular diseases. Image analysis with the help of AI in diagnosing infectious diseases has also contributed to the identification and classification of microbes [22]. Cardiology has also been a field in which ML has been applied to improve the diagnosis and treatment planning of cardiac amyloidosis [23]. While these examples highlight the multidisciplinary adoption of AI in medicine, the present review specifically focuses on its applications in digital pathology, computational pathology, and histopathological image analysis. Digital pathology combined with AI is another field in which nephropathology has benefited, improving the reproducibility and efficiency of diagnosis in nephropathologists [24,25]. Moreover, AI in digital pathology can be used in cancer staging, which demonstrates that this technology is becoming a part of the diagnostic work of contemporary pathology [26]. AI-based tools have demonstrated quantitative accuracy and promise to be implemented into clinical practice [27,28]. All these examples indicate how wide the field of AI is in modern diagnostics, covering oncology, neurology, cardiology, infectious diseases, and pathology.

Despite these developments, there are still problems. Some of the present limitations are a lack of data, absence of standardised validation systems, interpretability, and barriers to clinical integration [29,30]. Most AI models are based on single-institution data, which restricts the generalisation of the findings to a wide population. Also, the ethical issues of algorithmic bias, transparency, and the regulatory approval process still act as obstacles to clinical adoption [31]. Regardless of these shortcomings, all of the evidence to date points to the revolutionary nature of AI in pathology and diagnostic medicine. The present review, therefore, focuses specifically on the application of AI and ML in digital pathology, computational pathology, and histopathological image analysis.

Rationale and aim of the review

Considering the accumulated amount of evidence regarding the use of AI in the field of diagnostics, especially in pathology, there is a necessity to develop an integrated synthesis of the findings that covers both the opportunities and the issues. Although AI applications have been widely reviewed in fields such as oncology, radiology, and general medicine [27], comprehensive synthesis focusing specifically on diagnostic pathology and histopathological image analysis remains limited.

This review of literature, then, tries to evaluate the uses, constraints, and clinical implications of AI and ML in diagnostic pathology. By summarising the latest studies, the review attempts to shed light on the present condition of AI in the field of pathology, what the obstacles that exist to its further use are, and how the tools be able to shape the future of diagnostic medicine.

## Review

Methodology

Protocol and Reporting Standards

This systematic literature review was conducted in accordance with the Preferred Reporting Items for Systematic Reviews and Meta-Analyses (PRISMA) 2020 guidelines. The review methodology was developed with rigour, transparency, and reproducibility in mind. All the significant issues were discussed before the commencement of the review, including the objectives of the review, eligibility criteria, search strategy, data extraction procedures, and synthesis methods.

Eligibility Criteria

Eligibility criteria were established to ensure that included studies specifically involved human or digital pathology datasets relevant to diagnostic pathology, including histopathology, cytology, or tissue-based imaging, as these represented the primary population of interest. The use of ML and AI methodologies in diagnostic pathology, such as support vector machines, convolutional neural networks, and deep learning, was considered the primary intervention of interest. Comparators, where applicable, included traditional diagnostic procedures, expert manual interpretation, or alternative computational approaches. The desired outcomes included diagnostic accuracy, sensitivity, specificity, workflow efficiency, and broader clinical implications. Studies applying AI in medical specialties without direct relevance to digital pathology, histopathological image analysis, or computational pathology workflows were excluded to maintain the specific focus of the review. The types of studies considered eligible included meta-analyses, systematic reviews, and peer-reviewed original research articles. To preserve methodological integrity and scientific rigour, studies published in languages other than English, conference abstracts, editorials, commentaries, and grey literature were excluded.

Information Sources and Search Strategy

A comprehensive search was conducted across multiple electronic databases, including the Cochrane Library, Web of Science, IEEE (Institute of Electrical and Electronics Engineers) Xplore, PubMed, and Scopus, between January 2010 and August 2025. The search strategy combined Medical Subject Headings (MeSH) with free-text keywords to capture the diverse array of AI and ML applications in pathology. An example of a PubMed search query is as follows:

("artificial intelligence" OR "machine learning" OR "deep learning" OR "neural network" OR "computational pathology") AND ("diagnostic pathology" OR "histopathology" OR "digital pathology" OR "cytology" OR "tissue imaging") AND ("applications" OR "challenges" OR "clinical implications")

The search strategy was adapted for each database by modifying controlled vocabulary terms, Boolean operators, and field tags according to the indexing structure of the respective database. Filters were applied to restrict results to studies published between January 2010 and August 2025 and to articles written in English.

In addition, manual searches of reference lists from selected articles and related reviews were undertaken to identify additional eligible studies not captured by database queries. This supplementary screening process was conducted to ensure that potentially relevant studies not retrieved through the electronic database search were also considered for inclusion.

Study Selection

**Figure 1:**
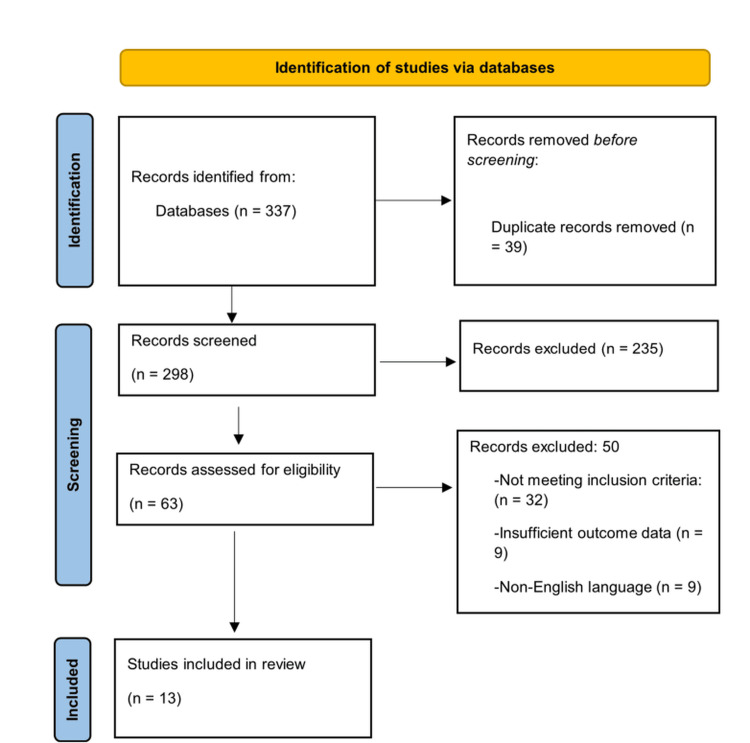
PRISMA chart showing the selection of the studies PRISMA: Preferred Reporting Items for Systematic Reviews and Meta-Analyses

A total of 337 records were identified through database searching. After the removal of 39 duplicate records, 298 unique records remained and were screened by title and abstract. Of these, 235 records were excluded during title and abstract screening because they did not meet the predefined inclusion criteria related to the study population, intervention, outcomes, or study design, leaving 63 full-text articles assessed for eligibility. Following full-text review, 50 articles were excluded, including 32 that involved ineligible study populations, interventions outside the scope of diagnostic pathology, or the absence of predefined diagnostic outcomes, nine with insufficient outcome data, and nine published in non-English languages. Finally, 13 studies met all eligibility criteria and were included in the qualitative synthesis. The relatively small number of included studies reflects the application of strict eligibility criteria restricting inclusion to studies that specifically applied AI or ML to digital pathology or histopathological image analysis and reported sufficient diagnostic performance outcomes. The process of selection is shown in the PRISMA 2020 flow diagram (Figure 1).

Data Extraction

A standardised data extraction form was used to gather data from the included studies in order to ensure uniformity and completeness. The extracted variables included bibliographic details (author, year, and country), characteristics of the dataset (size, type, and source of pathology images), AI/ML methodology (type of model, preprocessing techniques, and validation methods), comparator methods, and diagnostic performance outcomes such as accuracy, sensitivity, specificity, and area under the curve (AUC). Clinical implications, reported challenges such as model interpretability, dataset limitations, and ethical considerations, and details of clinical applications were also documented. Data extraction was conducted independently by two reviewers, and discrepancies were resolved through discussion or consultation with a third reviewer.

Quality Assessment

The methodological quality of the included studies was evaluated using validated appraisal tools appropriate to the different study designs. The assessment was conducted independently by two reviewers to ensure methodological consistency and reduce evaluation bias. Diagnostic accuracy studies were assessed using Quality Assessment of Diagnostic Accuracy Studies - version 2 (QUADAS-2) [30], predictive modelling studies using PROBAST (Prediction model Risk Of Bias ASsessment Tool) [31], and systematic reviews using AMSTAR-2 (A MeaSurement Tool to Assess Systematic Reviews 2) [32]. Any disagreements in quality ratings were resolved through discussion and consensus, with consultation from a third reviewer when necessary. The risk of bias of each study was considered during the synthesis and interpretation of the results and categorised as low, moderate, or high.

Data Synthesis

Due to substantial heterogeneity in datasets, AI/ML approaches, and reported outcomes, a narrative synthesis was considered the most appropriate methodology. The extracted data were synthesised thematically into three overarching areas: applications of AI/ML in diagnostic pathology, challenges and limitations reported in the literature, and clinical implications for the integration of AI/ML into pathology workflows. Where possible, results were summarised to facilitate cross-study comparison of diagnostic performance and methodological characteristics.

Results

Study Characteristics

The evidence base was mostly comprised of retrospective model-development studies with whole-slide images, and supplemented with multicenter cytology deep learning studies and a study on benchmarking reproducibility. The datasets were of moderate, as well as large, multi-source cohorts, including public repositories (including The Cancer Genome Atlas (TCGA)) and hospital-obtained data. In the literature, deep learning methods were used, but most often, convolutional neural networks, often with weakly supervised learning or transfer learning, to overcome small annotation quantities. External validation was also included in several studies, but prospective evaluation and real-world clinical deployment assessments were not common (Table 1).

**Table 1:** Key characteristics of included studies (N = 13) AI: Artificial Intelligence, ML: Machine Learning, DL: Deep Learning, WSI: Whole-Slide Image, HNSCC: Head and Neck Squamous Cell Carcinoma, TCGA: The Cancer Genome Atlas, H&E: Hematoxylin and Eosin, CNN: Convolutional Neural Network, AUC: Area Under the Curve, ROC: Receiver Operating Characteristic, TNM: Tumor-Node-Metastasis, IDH: Isocitrate Dehydrogenase, CUP: Cancer of Unknown Primary

Author (Year)	Domain of Pathology	Study Design	Dataset Characteristics	AI/ML Method	Key Findings	Challenges Reported
Dolezal et al. [10]	General digital pathology (multi-organ WSI benchmarking)	Framework development + multi-task benchmarking study	31 computational pathology tasks across publicly available WSI datasets including CAMELYON and TCGA subsets	Slideflow deep learning framework (CNN-based pipelines; WSI-level modeling)	Standardized WSI workflows; demonstrated scalability across tasks	Reproducibility issues; computational burden
Neidlinger et al. [11]	Multi-organ digital pathology	Benchmarking and reproducibility study	31 histopathology datasets covering classification, mutation prediction, survival prediction	Deep learning framework for efficient pathology image analysis	Identified variability in performance and generalizability gaps	Dataset heterogeneity; need for standardization
Kalra et al. [14]	Pan-cancer histopathology (TCGA)	Retrospective large-scale image retrieval study	>24,000 TCGA WSIs across multiple cancer types	Yottixel content-based image retrieval (deep feature embeddings)	Accurate pan-cancer image search and diagnostic consensus	Dependence on large annotated repositories
Lu et al. [19]	Multi-institutional computational pathology	Federated learning development study	Multi-institutional WSI datasets including TCGA and hospital cohorts; distributed training without data sharing	Federated deep learning with multi-instance learning	Privacy-preserving distributed model training with competitive accuracy	Communication overhead; site heterogeneity
Campanella et al. [20]	Prostate, breast, skin cancer pathology	Retrospective clinical-grade validation study	44,732 WSIs from 15,187 patients (NYU Langone Health)	Weakly supervised deep learning (MIL-based CNN)	Clinical-grade AUC ≥0.98 without pixel-level annotation	Single-institution dataset; limited external validation
Tauqeer et al. [22]	Breast cancer lymph node metastasis	Retrospective model development + validation	CAMELYON16 & CAMELYON17 datasets; 1,399 WSIs	Selective neighborhood attention-based CNN	AUC >0.99 for metastasis detection and staging	Dataset imbalance; domain adaptation
Wentzensen et al. [23]	Cytopathology (dual-stain cervical screening)	Algorithm development + multi-cohort validation	4,253 dual-stained cytology slides across screening cohorts	CNN-based whole-slide cytology classifier	Higher specificity with comparable sensitivity vs manual review	Platform-dependent variability
Kanavati et al. [24]	Cervical cytology (liquid-based WSI)	Pilot development + external testing	1,605 WSIs for training; 1,468 WSIs across three test sets	Deep CNN WSI classification model	AUC 0.89-0.96 for neoplastic detection	Workflow integration constraints
Tian et al. [25]	Cytology (Cancer of Unknown Primary - effusion samples)	Multicenter retrospective development + external validation	57,220 cytology images from 43,688 patients across four hospitals	TORCH deep learning cytology system	AUC 0.953-0.991; high tumor-origin prediction accuracy	Need for prospective randomized validation
Yu et al. [26]	Head & neck squamous cell carcinoma	Retrospective WSI-based model development with patient-level split	791 frozen H&E WSIs from 500 TCGA-HNSC patients; 80/20 split	ResNet34 transfer learning (MIL aggregation)	AUC 0.998 tumor detection; 0.992 stage prediction	Frozen sections only; limited FFPE validation
Challa et al. [27]	Breast cancer lymph node metastasis	Clinical workflow validation study	Multi-center digitized breast lymph node WSIs; several thousand annotated slides	AI-assisted diagnostic workflow model	Improved diagnostic efficiency in digital workflow	Regulatory and implementation considerations
Zuo et al. [28]	Neuropathology (glioma classification)	Weakly supervised retrospective study + external validation	TCGA glioma WSIs + independent hospital cohort	Weakly supervised CNN with attention pooling	Accurate glioma subtype classification	Limited annotated external datasets
Fell et al. [29]	Head & neck cancer staging	Retrospective model development + patient-level validation	791 TCGA frozen WSIs (500 patients)	Inception-ResNet34 CNN	High AUC (>0.99) for TNM stage prediction	Generalizability concerns

Data Scale and Validation Patterns

**Figure 2:**
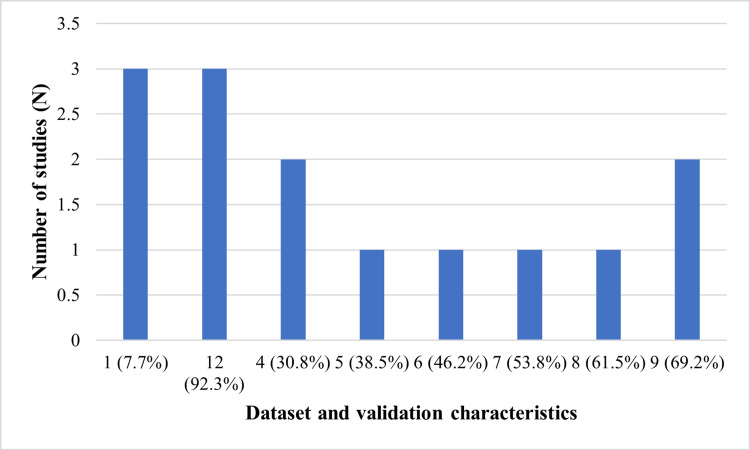
Distribution of dataset and validation characteristics across included studies (N = 13)

The incorporated studies consisted of studies that were remarkably diverse in sources of data and methods of validation. Some of the studies used repositories that are publicly available, and some used nothing but institution-derived data, which showed some variation in accessibility, case mix, and annotation practices. There was also a variety of methods of validation, as some of the studies used independent external cohorts to validate the performance, and others used performance within internal splits, where inference about the generalizability is limited. Both single and multicenter datasets were present, which shows the current trend of moving to wider data aggregation in computational pathology. The most used methodological paradigm was deep learning, and weakly supervised and transfer learning were commonly used to overcome the lack of annotations, whereas privacy-preserving federated learning was not widespread. Figure 2 presents the count of datasets and validation characteristics in the included studies.

Risk of Bias Assessment

The QUADAS-2 was used to assess the risk of bias and was designed to assess bias in four domains, namely patient selection, index test, reference standard, and flow and timing. The majority of studies were retrospective and based on secondary data, which led to some concerns about patient selection and applicability. It is the index test domain that was considered to be of low risk, generally because of well-documented model architectures and performance reporting, but sometimes lacked the details about preprocessing pipelines and data partitioning. The reference standards were largely grounded on expert annotations of pathologists, which minimised the bias in measurements. Flow and timing were mostly suitable in retrospective designs, but prospective validation was seldom done. In general, the majority of the studies were associated with a moderate risk of bias, which was mainly predetermined by retrospective design and the lack of external validation. Table 2 displays risk-of-bias ratings for every study included.

**Table 2:** Risk of bias assessment of included studies using the QUADAS-2 tool (N = 13) QUANDAS-2: Quality Assessment of Diagnostic Accuracy Studies - version 2

Study	Patient Selection	Index Test	Reference Standard	Flow and Timing	Overall Risk
Dolezal et al. [10]	Low	Low	Low	Low	Low
Neidlinger et al. [11]	Low	Low	Low	Low	Low
Kalra et al. [14]	Moderate	Moderate	Moderate	Moderate	Moderate
Lu et al. [19]	Moderate	Moderate	Moderate	Moderate	Moderate
Campanella et al. [20]	Moderate	Low	Low	Moderate	Moderate
Tauqeer et al. [22]	Moderate	Low	Moderate	Moderate	Moderate
Wentzensen et al. [23]	Moderate	Low	Low	Moderate	Moderate
Kanavati et al. [24]	Moderate	Low	Moderate	Moderate	Moderate
Tian et al. [25]	Moderate	Low	Low	Moderate	Moderate
Yu et al. [26]	Moderate	Low	Moderate	Moderate	Moderate
Challa et al. [27]	Moderate	Low	Low	Moderate	Moderate
Zuo et al. [28]	Moderate	Low	Moderate	Moderate	Moderate
Fell et al. [29]	Low	Low	Low	Low	Low

PROBAST and AMSTAR-2 Assessment

PROBAST was applied to studies primarily focused on predictive modelling outcomes, and most were judged to have a moderate overall risk of bias due to retrospective design, limited external validation, and incomplete reporting of preprocessing and data partitioning. AMSTAR-2 was intended for systematic reviews; however, the final included set (n = 13) consisted predominantly of original model-development and validation studies, making the AMSTAR-2 assessment not applicable for most included studies.

Discussion

This systematic review integrated findings from 13 studies evaluating the use of AI and ML in diagnostic pathology. The studies were largely retrospective model development and validation studies based on whole-slide imaging and cytology samples. Deep learning models, especially convolutional neural networks and weakly supervised multi-instance learning models, were the primary methodological strategies. Excellent diagnostic accuracy was found to be achieved for tumour detection, classification, and staging in controlled validation studies. The applications covered breast pathology, cytopathology, neuropathology, head and neck oncology, and multi-organ computational pathology, which were shown to be technically feasible in various subspecialties. External validation was performed in some studies, but prospective workflow validation studies were rare. Risk of bias was found to be moderate in most studies, mainly due to retrospective study design and applicability.

These results are consistent with current trends in clinical-grade computational pathology systems that use weakly supervised deep learning on whole-slide images [20]. Reproducibility benchmarking studies have emphasised the variability of results across datasets and institutions, underlining the need for standardised validation approaches [29]. Federated learning approaches have shown the potential for privacy-preserving multi-institutional model training in computational pathology settings [19]. Appraisal tools like QUADAS-2 offer a conceptual framework for evaluating bias in studies of diagnostic accuracy that involve AI models [30]. PROBAST also facilitates the systematic assessment of predictive modelling studies by addressing applicability and overfitting issues [31]. AMSTAR-2 sets quality standards when systematic reviews are used in evidence synthesis in healthcare studies [32].

The incorporation of AI technology into the diagnostic process represents a broader shift that is also taking place in other image-based specialities of medicine. Image-based AI applications in dermatological diagnosis illustrate the potential of visual pattern recognition models to assist in the interpretation of complex inflammatory disease processes [33]. Similarly, AI-based classification models in dermatology illustrate the expansion of deep learning models beyond the domain of cancer-focused applications [34]. Non-generative models of AI in medicine highlight the importance of decision-support augmentation rather than replacement by AI technology [35]. Applications in oncology research further illustrate the contribution of AI to biomarker stratification and trial optimisation models [36]. In neuro-oncology, AI models have been investigated for glioblastoma screening and classification, illustrating the domain-specific adaptability of deep learning models [37]. ML-based tissue cytometry in nephropathology illustrates the translational expansion of AI models into renal disease diagnosis [38]. AI in surgical pathology illustrates the increasing integration of computational models into routine histopathological interpretation processes [39].

The evidence suggests that AI systems are capable of achieving high technical accuracy on a range of pathology subspecialties within the context of structured experimental settings. The availability of digitised histopathology databases, computational infrastructure, and methodological frameworks has facilitated the development and validation of models. AI systems thus serve as an assistive decision-support tool within digital pathology platforms, improving efficiency and remaining grounded in expert pathological interpretation.

Limitations of the Review

The limitations of this review are inadequate access to large, well-annotated datasets in the studies incorporated in this review, limiting the extrapolation of the results. The quality of evidence to be assessed was also minimised by the fact that AI models with low interpretability are relied on. Reporting inconsistencies regarding the workflow integration, technical requirements, and institutional readiness also restricted the assessment of the practical implementation obstacles. Also, regulatory and ethical concerns, including approvals, accountability, and algorithmic bias, were frequently not sufficiently covered in the original research, limiting the level of coverage in these fields.

Future Recommendations

Each new investigation of AI and ML in the field of diagnostic pathology requires future researchers to build large, diverse, multi-institutional datasets that increase the external validity of algorithms and minimise bias. Shared data-sharing models and standardisation of imaging procedures are significant towards achieving consistency of model performance across institutions. It should be stressed that the development of explainable AI is required to make AI more interpretable, increase clinician trust, and assist with transparent decision-making. The everyday integration of the workflow demands spending on digital infrastructure, extensive training of pathologists, and the creation of user-friendly platforms that supplement the ones used in laboratories. In addition to this, it has to be validated externally in real-world, prospective studies in order to be clinically reliable. The regulatory bodies must work in close association with the researchers to give clear avenues of approval and ethical standards on matters of accountability, patient safety, and algorithmic fairness. Lastly, a cross-functional team of computer scientists, pathologists, and policymakers will be crucial in transforming AI innovations into safe, scalable, and equitable diagnostic solutions to find the future of pathology.

## Conclusions

This systematic review demonstrates that AI and ML applications in diagnostic pathology achieve high diagnostic accuracy across multiple subspecialties under retrospective validation conditions. Deep learning-based systems show consistent performance in tumour detection, classification, and staging tasks using whole-slide imaging and cytology datasets. Although external validation was undertaken in selected studies, prospective clinical workflow evaluation remains limited. Risk-of-bias assessments indicate a moderate overall methodological risk, primarily related to the retrospective design and patient selection. Current evidence supports the role of AI as an adjunctive decision-support tool within digital pathology environments. Continued methodological rigour and structured validation will determine the extent to which these systems are integrated into routine clinical practice.

## References

[1] Ahmed N, Abbasi MS, Zuberi F, Qamar W, Halim MS, Maqsood A, Alam MK (2021). Artificial intelligence techniques: analysis, application, and outcome in dentistry-a systematic review. Biomed Res Int.

[2] Chen M, Copley SJ, Viola P, Lu H, Aboagye EO (2023). Radiomics and artificial intelligence for precision medicine in lung cancer treatment. Semin Cancer Biol.

[3] Chassagnon G, De Margerie-Mellon C, Vakalopoulou M, Marini R, Hoang-Thi TN, Revel MP, Soyer P (2023). Artificial intelligence in lung cancer: current applications and perspectives. Jpn J Radiol.

[4] Majumder A, Sen D (2021). Artificial intelligence in cancer diagnostics and therapy: current perspectives. Indian J Cancer.

[5] Chang CH, Lin CH, Lane HY (2021). Machine learning and novel biomarkers for the diagnosis of Alzheimer's disease. Int J Mol Sci.

[6] Chen X, Wang X, Zhang K (2022). Recent advances and clinical applications of deep learning in medical image analysis. Med Image Anal.

[7] Rabbani N, Kim GY, Suarez CJ, Chen JH (2022). Applications of machine learning in routine laboratory medicine: current state and future directions. Clin Biochem.

[8] Qi YJ, Su GH, You C, Zhang X, Xiao Y, Jiang YZ, Shao ZM (2024). Radiomics in breast cancer: current advances and future directions. Cell Rep Med.

[9] Bedrikovetski S, Dudi-Venkata NN, Kroon HM (2021). Artificial intelligence for pre-operative lymph node staging in colorectal cancer: a systematic review and meta-analysis. BMC Cancer.

[10] Dolezal JM, Kochanny S, Dyer E (2024). Slideflow: deep learning for digital histopathology with real-time whole-slide visualization. BMC Bioinformatics.

[11] Neidlinger P, Lenz T, Foersch S (2025). A deep learning framework for efficient pathology image analysis. arXiv.

[12] Zheng X, He B, Hu Y (2022). Diagnostic accuracy of deep learning and radiomics in lung cancer staging: a systematic review and meta-analysis. Front Public Health.

[13] Yin H, Xie J, Xing S (2024). Machine learning-based analysis identifies and validates serum exosomal proteomic signatures for the diagnosis of colorectal cancer. Cell Rep Med.

[14] Kalra S, Tizhoosh HR, Shah S (2019). Pan-cancer diagnostic consensus through searching archival histopathology images using artificial intelligence. arXiv.

[15] Balkenende L, Teuwen J, Mann RM (2022). Application of deep learning in breast cancer imaging. Semin Nucl Med.

[16] Huang B, Sollee J, Luo YH (2022). Prediction of lung malignancy progression and survival with machine learning based on pre-treatment FDG-PET/CT. EBioMedicine.

[17] Turkbey B, Haider MA (2022). Deep learning-based artificial intelligence applications in prostate MRI: brief summary. Br J Radiol.

[18] Wang H, Minnema J, Batenburg KJ, Forouzanfar T, Hu FJ, Wu G (2021). Multiclass CBCT image segmentation for orthodontics with deep learning. J Dent Res.

[19] Lu MY, Kong D, Lipkova J (2020). Federated learning for computational pathology on gigapixel whole slide images. arXiv.

[20] Campanella G, Hanna MG, Geneslaw L (2019). Clinical-grade computational pathology using weakly supervised deep learning on whole slide images. Nat Med.

[21] Allegra A, Mirabile G, Tonacci A, Genovese S, Pioggia G, Gangemi S (2023). Machine learning approaches in diagnosis, prognosis and treatment selection of cardiac amyloidosis. Int J Mol Sci.

[22] Tauqeer A, Asif A, Sadeghi-Naini A (2025). Detection, localization, and staging of breast cancer lymph node metastasis in digital pathology whole slide images using selective neighborhood attention-based deep learning. Sci Rep.

[23] Wentzensen N, Lahrmann B, Clarke MA (2021). Accuracy and efficiency of deep-learning-based automation of dual stain cytology in cervical cancer screening. J Natl Cancer Inst.

[24] Kanavati F, Hirose N, Ishii T, Fukuda A, Ichihara S, Tsuneki M (2022). A deep learning model for cervical cancer screening on liquid-based cytology specimens in whole slide images. Cancers (Basel).

[25] Tian F, Liu D, Wei N (2024). Prediction of tumor origin in cancers of unknown primary origin with cytology-based deep learning. Nat Med.

[26] Yu H, Yu W, Enwu Y, Ma J, Zhao X, Zhang L, Yang F (2025). Enhancing head and neck cancer detection accuracy in digitized whole-slide histology with the HNSC-classifier: a deep learning approach. Front Mol Biosci.

[27] Challa B, Tahir M, Hu Y (2023). Artificial intelligence-aided diagnosis of breast cancer lymph node metastasis on histologic slides in a digital workflow. Mod Pathol.

[28] Zuo M, Xing X, Zheng L (2025). Weakly supervised deep learning-based classification for histopathology of gliomas: a single center experience. Sci Rep.

[29] Fell C, Mohammadi M, Morrison D, Arandjelovic O, Caie P, Harris-Birtill D (2022). Reproducibility of deep learning in digital pathology whole slide image analysis. PLOS Digit Health.

[30] Whiting PF, Rutjes AW, Westwood ME (2011). QUADAS-2: a revised tool for the quality assessment of diagnostic accuracy studies. Ann Intern Med.

[31] Wolff RF, Moons KG, Riley RD (2019). PROBAST: a tool to assess the risk of bias and applicability of prediction model studies. Ann Intern Med.

[32] Shea BJ, Reeves BC, Wells G (2017). AMSTAR 2: a critical appraisal tool for systematic reviews that include randomised or non-randomised studies of healthcare interventions, or both. BMJ.

[33] Goessinger EV, Gottfrois P, Mueller AM, Cerminara SE, Navarini AA (2024). Image-based artificial intelligence in psoriasis assessment: the beginning of a new diagnostic era?. Am J Clin Dermatol.

[34] Cao F, Yang Y, Guo C, Zhang H, Yu Q, Guo J (2025). Advancements in artificial intelligence for atopic dermatitis: diagnosis, treatment, and patient management. Ann Med.

[35] Pantanowitz L, Pearce T, Abukhiran I (2025). Nongenerative artificial intelligence in medicine: advancements and applications in supervised and unsupervised machine learning. Mod Pathol.

[36] Azenkot T, Rivera DR, Stewart MD, Patel SP (2025). Artificial intelligence and machine learning innovations to improve design and representativeness in oncology clinical trials. Am Soc Clin Oncol Educ Book.

[37] Brown ED, Pelcher I, Leon S, Karkare AN, Barbero JA, Ward M, Schulder M (2025). Artificial intelligence applications in the screening and classification of glioblastoma. J Neurosurg Sci.

[38] Winfree S, Al Hasan M, El-Achkar TM (2022). Profiling immune cells in the kidney using tissue cytometry and machine learning. Kidney360.

[39] Sagiv C, Hadar O, Najjar A, Pahnke J (2025). Artificial intelligence in surgical pathology - where do we stand, where do we go?. Eur J Surg Oncol.

